# Public and Healthcare Professionals’ Knowledge and Attitudes toward Binge Eating Disorder: A Narrative Review

**DOI:** 10.3390/nu9111267

**Published:** 2017-11-21

**Authors:** Deborah Lynn Reas

**Affiliations:** 1 Regional Department for Eating Disorders, Division of Mental Health and Addiction, Oslo University Hospital, N-0424 Oslo, Norway; deborah.lynn.reas@ous-hf.no; Tel.: +47-2301-6323; 2 Institute of Psychology, Faculty of Social Sciences, University of Oslo, N-0317 Oslo, Norway

**Keywords:** binge eating disorder, stigma, attitudes, knowledge, healthcare professionals, public, mental health literacy

## Abstract

Binge eating disorder (BED) is characterized by recurrent binge eating and marked distress in the absence of inappropriate compensatory behaviors for weight control. BED is prevalent in men and women, is associated with elevated psychosocial and functional impairment, and is associated strongly with obesity and related medical comorbidities. The aim is to provide a brief, state-of-the-art review of the major and recent findings to inform educational and awareness campaigns, stigma reduction interventions, as well as current clinical practice and future research. A narrative approach was used to synthesize emerging literature on the public and healthcare professionals’ knowledge and attitudes toward individuals with BED in comparison to other eating disorders (EDs) or mental illness. A total of 13 articles were reviewed. Nine studies investigated community samples and four studies investigated healthcare professionals. The reviewed literature suggested that BED is perceived by the public as less impairing, less severe, and “easier-to-treat” than other EDs. Attitudes and beliefs reflecting perceived blameworthiness and lack of self-discipline were ascribed to vignettes with BED. Community studies indicated a low level of public awareness that BED constitutes a discreet eating disorder. The literature on healthcare professionals’ knowledge and attitudes toward BED remains very limited. The few existing studies suggest encouraging trends in recognition and diagnostic accuracy, yet there remains a need for increased clinical awareness of BED-associated medical complications and knowledge of full BED diagnostic criteria.

## 1. Introduction

Binge eating disorder (BED) is formally recognized in the fifth edition of the Diagnostic and Statistical Manual of Mental Disorders (DSM-5) [1] as an eating disorder. It is defined by recurrent binge eating (i.e., discrete episodes of objective overeating while experiencing a subjective loss of control during the eating), marked distress about binge eating, and the absence of inappropriate weight compensatory behaviors (e.g., self-induced vomiting, laxative misuse, extreme exercise, or extreme food restriction). Diagnostic criteria specify that binge eating episodes must occur at least once weekly for 3 months, and be associated with three (or more) of the following: eating much more rapidly than normal, eating until uncomfortably full, eating large amounts of food when not feeling physically hungry, eating alone because of feeling embarrassed by how much one is eating, and feeling disgusted with oneself, depressed, or very guilty afterwards [1]. Binge eating disorder is not “new”, as it was historically described in the literature in the 1950’s by Albert Stunkard [2], who reported a 30-year old male who ate “enormous amounts of food in relatively short periods, followed by severe discomfort and expressions of self-condemnation” (p. 289). The inclusion of BED as a research category in the Criteria Sets and Axes Provided for Further Study of the fourth edition of the Diagnostic and Statistical Manual of Mental Disorders (DSM-IV) [3] stimulated considerable research supporting its clinical distinctiveness and validity as a diagnostic entity [4,5,6,7]. 

BED is associated with significantly elevated risk of medical and psychiatric comorbidity [8], lower quality of life, and impairment in functioning and work productivity [9]. The degree of role impairment in BED due to eating disorder symptomology is similar to the degree of impairment found in bulimia nervosa (BN) [10,11]. In terms of psychiatric comorbidity, the World Health Organization (WHO) surveys found that 80% of individuals with BED had met lifetime criteria for at least one other DSM-IV disorder, and subsequent studies suggest the degree of psychiatric comorbidity is linked to severity of binge eating [12] or eating disorder psychopathology [13], rather than body mass index (BMI). The highest rates of psychiatric comorbidity in a sample of 11,588 adult men and women presenting to eating disorder (ED) treatment clinics in Sweden were found among women with BED as compared to women with anorexia nervosa (AN) or BN [14]. Consistent with these findings, in a consecutive series of 404 treatment-seeking patients with BED, 73.8% had at least one additional lifetime psychiatric disorder, most commonly mood (54.2%), anxiety (37.1%), and substance use (24.8%) [15]. 

Although BED can occur across the BMI spectrum, it is significantly associated with risk of obesity and related comorbidities [11,16,17]. Data from the National Comorbidity Survey Replication (NCS-R) indicated that 42% of patients meeting the DSM-IV criteria for BED were classified with obesity [10]. Obesity, however, is not a diagnostic requirement for BED. Among US adults with obesity (BMI ≥ 30 kg/m^2^) in the Collaborative Psychiatric Epidemiology Surveys, the prevalence of BED was 5.5% in women 2.9% in men [18]. Evidence strongly suggests that among people with obesity, those meeting the DSM-5 BED diagnostic criteria constitute a meaningful and distinct subgroup [19], with a greater psychiatric illness burden [20]. 

Global data indicate that BED is more prevalent than anorexia nervosa (AN) and bulimia nervosa (BN) [10,11]. The WHO World Mental Health Surveys of 14 countries (*N* = 24,124 adults) found a total lifetime prevalence rate of 1.9% [11]. The highest country-specific lifetime prevalence estimates were found in Brazil (4.7%), followed by the US (2.6%) and Portugal (2.4%) [11]. Compared to AN and BN, the gender and age distribution of BED is less skewed, with comparable distribution between racial/ethnic groups across the adult lifespan [10,21]. Men are estimated to comprise approximately 40% of cases [10,16]. Age of onset of BED is slightly older than AN or BN, typically occurring during late adolescence or young adulthood (e.g., median onset = 23.3 years) [11], although infrequent or subthreshold binge eating behavior is not uncommon in children or adolescents [22]. Despite onset in late adolescence or young adulthood, the mean age of enrollment in treatment trials is mid-40s [23], indicating a lengthy delay of adequate treatment. Indeed, only a small proportion of individuals with BED is ever diagnosed and treated [24,25]. Only 3.2% of the respondents in the National Health and Wellness Survey who fulfilled the DSM-5 criteria for BED in the past 12 months reported they had ever received a formal diagnosis [10]. In a study of primary care and obstetric gynecology patients (*N* = 4651), fewer than one in ten cases had been recognized by their physician [26]. Collectively, these data indicate a substantial unmet need in the recognition and diagnosis of BED that must be addressed to successfully provide treatment services to those who may benefit. 

Insufficient knowledge and negative attitudes toward eating disorders have been identified as potential reasons for inadequate detection or delayed treatment for EDs [27,28,29]. Stigma is a well-recognized obstacle to help-seeking and the provision of effective care for EDs [30]. Indeed, a literature review found that stigma is the most frequently identified barrier to help-seeking by individuals with eating disorders [30]. Stigma adds to the burden of eating disorders and has numerous adverse effects on quality of life and mental health, including worsening eating disorder pathology [31]. Individuals afflicted by other EDs such as AN or BN often face depreciatory societal stereotypes, including characterizations as attention-seeking, or as having a trivial, self-imposed problem [32,33]. One in three members of the public stigmatize individuals with AN and BN as “having only themselves to blame” and feel they should just be able to “pull themselves together”, whereas fewer than 1 in 10 members of the public stigmatize schizophrenia in this way [34]. Furthermore, EDs are often viewed as afflictions of “white, young females” to the exclusion of diverse presentations [31,35]. 

Healthcare professionals are not immune to harboring negative stereotypes of ED patients (e.g., “vain”, “manipulative”) [36]. Prevailing views among clinicians may either facilitate early detection or enable rapid access to care, or, conversely, undermine help-seeking behavior or therapeutic engagement [37]. Traditional or female-centric conceptualizations of ED, in addition to pejorative language used in earlier diagnostic criteria (i.e., “refusal to maintain weight”) [3] as well as the treatment literature (e.g., “difficult-to-treat”), have arguably upheld stigmatizing or stereotypical beliefs within the ED field itself. Negative clinician reactions toward individuals with ED (e.g., feelings of frustration, anger, and hopelessness) are associated stigmatizing beliefs and patients’ lack of improvement (see [38] for a review). Research on patient perspectives in AN suggests that patients highly value attributes such as acceptance, empathy, warmth, and openness by health professionals [39]. Similarly, individuals with BED value safe, non-judgmental interactions with healthcare providers [40] and strongly prefer that therapists use neutral terms when discussing eating and weight [41,42]. 

The stigmatization of BED has received particularly little attention relative to stigma associated with obesity or the other eating disorders, yet this field of study is emerging and timely. From the outset, the recognition of binge eating disorder as a formal psychiatric diagnosis constituted a major revision to the diagnostic system for feeding and eating disorders [43], yet its inclusion in the DSM was met with some skepticism. The former chairman of the DSM-IV taskforce, Allen Francis, MD, has been an outspoken critic of recognizing BED as a formal diagnosis in DSM-5, contending that the disorder was “fake” and represented no more than “gluttony” [44]. Francis and Widiger [45] pinpointed BED as one of the new DSM-5 diagnoses which may “potentially create false epidemics of misidentified pseudopatients” (p. 122). Despite these initial concerns of diagnostic hyperinflation following the inclusion of BED in the DSM-5 [44,45], ensuing research has found little evidence that diagnostic criteria have “overpathologized” normative eating patterns among normal or overweight individuals [46,47,48]. It is yet unclear, however, the extent to which stereotypes or misperceptions of BED are widespread in the community-at-large or among healthcare professionals.

## 2. Aim

The present review summarizes the existing literature on the stigmatization of binge eating disorder (BED) by examining studies of public and healthcare professionals’ knowledge and attitudes toward binge eating disorder. To date, no such review exists. The aim is to provide a brief state-of-the-art review of the major and recent findings to inform current clinical practice, awareness campaigns, and future research, with an emphasis on the developments since the publication of the DSM-5 (APA, 2013). Studies investigating weight-related stigma are not considered, as this literature has been thoroughly reviewed elsewhere [29,49,50,51]. The reader is also referred to excellent reviews summarizing the evidence related to the effects of weight stigma on eating behavior [49,51]. This literature has consistently shown associations between frequency of exposure to weight stigma, including weight bias internalization and risk of engaging in binge or “loss of control” eating. The present paper contributes uniquely to the literature providing a review of studies assessing lay and healthcare professionals’ knowledge and attitudes toward BED, including beliefs about the causes and comparisons of stigmatizing attitudes toward BED relative to other mental disorders.

## 3. Method

A narrative approach was used to synthesize studies of the general public or healthcare professionals that examined attitudes, knowledge, or perceptions specifically related to BED. An electronic search was performed for all English-language articles of social science and medical databases including PsychINFO and MEDLINE via PubMed to 1 October 2017. Keyword searches included various combinations of “binge eating disorder” or “binge eating” and “stigma,” “attitudes”, “knowledge”, “perceptions”, and “mental health literacy.” Reference lists were manually searched and electronic search tools were utilized such as “cited by” and “related to” to identify literature not found by the main search. A total of 13 articles were reviewed. Study characteristics and main findings related to BED were extrapolated and summarized in Table 1 and Table 2. The vast majority (12 of 13) of studies utilized a case vignette methodology. Four studies [52,53,54,55] investigated healthcare professionals, and nine studies investigated samples from the community [56,57,58,59,60,61,62]. Of the community studies, five were university samples of undergraduates [56,57,58,61,63], two were general population health surveys [59,62], and two investigated adolescents [60,64]. A brief description of these studies followed by a discussion is provided below to serve as a comprehensive resource for further investigations. 

## 4. Results

### 4.1. Community Studies of Knowledge and Attitudes toward BED

As part of the 2005 South Australian Health Survey, Mond and Hay [59] randomly selected 1031 individuals (61% female) to participate in a BED mental health literacy survey. A fictional case vignette was read aloud to participants describing a 32-year old female with obesity who engaged in binge eating. Following the vignette, participants were forced to select 1 of 12 responses that they felt was the character’s “main problem.” Approximately one-third of participants rated “depression” as the main problem, and one-third endorsed low self-esteem/lacks self-confidence (28.3%), whereas only 11.7% endorsed BED as the main problem. General physicians (25%) and dietician/nutritionists (24.5%) were viewed as the type of treatment providers most likely to deliver effective treatment. Vitamins and minerals were viewed most favorably as potentially effective treatment options. Approximately 21% of participants rated behavioral weight loss to likely be the most helpful treatment for binge eating, and 19.5% felt that “getting information about the problem and available services” would be the most helpful. Only 4.6% of participants felt that cognitive behavior therapy would be the most helpful treatment. 

In a study by Bannon et al. [56], undergraduate students (*N* = 376) were assigned randomly to read one of six scenarios depicting a woman with obesity either with or without the presence of binge eating. Participants assigned to the obesity with binge eating condition rated the character more negatively, as having a worse prognosis, and being more likely to drop-out of treatment than a woman with obesity without BED. The fictional woman who engaged in binge eating was rated as less attractive and more blameworthy, and respondents desired greater social distance. Men held significantly more stigmatizing attitudes than women across the domains of physical and romantic attractiveness, social disparagement, and negative judgments. Psychotherapy or drug-therapy combination treatment was perceived as the most beneficial treatments for BED.

Ebneter et al. [58] investigated the relationship between “just world” beliefs (i.e., people get what they deserve in life) and stigmatizing attitudes toward ED and obesity. Participants were 447 university students assigned randomly to read a vignette depicting a character with AN, BN, BED, or obesity. Assessments of stigmatizing attitudes, just world beliefs, causal beliefs, and acquaintance with the condition depicted in the vignette were measured. Findings showed that that stronger beliefs in a just world and causal attributions to parenting and a lack of self-discipline were positively correlated with stigma toward AN, BN, BED, and obesity (rs = 0.35 to 0.41). Participants who were acquainted with someone with BED showed a similar level of stigma, lending little support to the “contact hypothesis” [65], which predicts that knowing someone affected by mental illness reduces prejudice. 

A study by Ebneter and Latner [57] compared stigmatizing attitudes across AN, BN, BED, obesity, and major depressive disorder (MDD). Over 400 university undergraduates viewed case vignettes, followed by an assessment of stigmatizing attitudes related to blameworthiness and impairment/distrust. Lack of self-discipline was perceived to contribute more to the development of BED and obesity than to the development of AN, BN, or MDD. The target with BED was blamed more than other EDs or depression, and was considered less impaired. The target with MDD was rated as the most impaired and least personally responsible for their illness, whereas the vignette with obesity was rated the most personally responsible for their illness. Of note, the target with obesity and BED was held less personally responsible than the vignette with obesity but without BED. 

Star et al. [62] investigated perceived discrimination toward an underweight female with AN, a normal-weight male with atypical ED, and a female with obesity and BED. Participants (*N* = 3047) were asked, “Do you think that (name of character) would be discriminated against by others in the community if they knew about her problem, for example, by an employer, a colleague, a family member, or by a health professional?” Results showed that the majority (66%) of participants believed the vignette with obesity and BED would be discriminated against, compared to 48% for the AN vignette, and 35% for the male vignette with atypical ED. Of participants who believed discrimination would occur, the vast majority (84%) believed that such discrimination would be weight-related, not eating-related. A higher proportion of older participants (aged 35–54) than younger participants (under 35) believed the vignette with comorbid obesity and BED would be discriminated against (58% vs. 71%, respectively). Notably, this age-related trend was not specific to BED, as younger participants were also less likely to believe discrimination would occur against the AN and atypical ED vignettes. 

Anderson et al. [64] recruited 1670 (68% female) adolescents aged 12–18 years from secondary schools as part of a mental health literacy survey in Australia. A vignette was presented that described either a female character with BED or BN. A higher percentage of respondents correctly identified BED than BN. Specifically, 28% and 43% of boys and girls, respectively, accurately recognized BED compared to 15% and 33% for BN. Three times as many boys perceived “lack of will-power” as the main problem for BED than for BN. Among girls, twice as many perceived “lack of will-power” as the main problem for BED than BN. Approximately 50% agreed or strongly agreed that BED is a serious problem. Still, in comparison to BN, a lower proportion of both boys and girls felt BED was a serious problem and required professional treatment. Overall, boys were more likely to perceive BED as less serious and attributable to lack of will power/self-control than girls.

A study by Murakami, Essayli, and Latner [63] investigated the relative stigmatization of eating disorders and obesity using male and female vignettes. A total of 318 undergraduates were randomly assigned to read case vignettes depicting either a male or female character diagnosed with AN, BN, BED, or obesity without BED. Stigmatizing attitudes and perceived mental health of the vignette were analyzed according to the target gender and target diagnosis. Targets with obesity were held more personally responsible compared to targets with AN, while BN, BED, and obesity were found similarly blameworthy for their conditions. Those with BED were rated as less impaired than targets with AN and BN, yet more impaired than targets with obesity without BED. The vignette with BN was more negatively judged and elicited more discomfort with proximity compared to the vignette with BED. No main effects for vignette gender were found.

A nationwide survey by O’Conner et al. [60] of 290 Irish adolescents aged 15–19 years old was conducted to assess ED literacy and attitudes in secondary schools. Participants were randomly assigned to read one of five vignettes depicting AN, BN, BED, type 1 diabetes, or depression. A series of questions assessed illness recognition, beliefs regarding illness duration, level of personal control over the illness, treatment efficacy, and potential causes of the illness, as well as participants’ own anticipated emotional reaction to interacting with the target. None of the participants correctly recognized the BED vignette. Symptoms of depression were more frequently correctly recognized (by 39.5% of the participants) than those of diabetes or ED. Forty percent of participants causally attributed BED to “self-control problems”, whereas less than 2% of participants causally attributed self-control problems to AN, depression, or diabetes (4.2% for BN). The vignette with BED was ascribed fewer positive personality traits than the vignettes with depression or AN. Despite this, young persons anticipated significantly more positive emotional reactions when interacting with the vignette with BED than the vignette with depression. 

Simpson and Mazzeo [61] examined young adults’ attitudes toward orthorexia nervosa relative to DSM-5 eating disorders. Orthorexia is defined as a pathological preoccupation with consuming healthy food, and strict avoidance of food was considered unhealthy. A total of 505 university students were randomly assigned to case vignettes depicting AN, BN, BED, or orthorexia. Participants thereafter responded to a series of questions related to causal beliefs, stigmatizing attitudes (attention-seeking, blameworthiness, self-attribution, responsibility, and fear and exclusion), perceptions of severity and desirability, and personality and behavioral attributes. Individuals with BED were viewed as significantly less “hard to talk to” and less “dangerous” than cases with orthorexia or AN. Individuals with BED were also viewed as more likely to “pull themselves together if they wanted to” than those with orthorexia. BED was considered “easier-to-treat” than AN or BN. Participants viewed “self-discipline” as a more important contributor to the development of BED than the other conditions. Participants believed that biological factors contributed *less* to the development of BED than orthorexia and AN. Negative personality characteristics were ascribed to individuals of all ED types, yet no significant group differences were found. Male respondents perceived all types of EDs to be less severe, and characterized EDs as more attention-seeking and blameworthy than female respondents.

### 4.2. Healthcare Professionals’ Knowledge and Attitudes toward BED

An earlier study by Crow et al. investigated physicians’ assessment and treatment practices for binge eating and obesity [53]. The sample was cross-disciplinary and consisted of 272 physicians, of whom 43.8% were in family practice, 35.7% in internal medicine, 13.6% obstetrician-gynecologist OB-GYN, and 6.9% other/missing. More than 40% of physicians reported that they had never assessed for binge eating, and an additional 42.8% estimated that 0 to 20% of their patients with obesity engaged in binge eating behavior. 

McNicholas et al. [54] conducted a web-based survey investigating knowledge and attitudes toward ED among Irish healthcare professionals (*N* = 171), including therapists/counsellors (40%), psychiatrists (20%), general physicians (15%), and psychologists (14%). Five vignettes depicted a fictional young person who presented with classic symptoms of AN, BN, BED, depression, or type 1 diabetes, followed by a series of questions regarding the perceived diagnosis, illness perceptions, anticipated outcome, feelings about treatment interactions, and target character gender. Only 19% accurately diagnosed BED as the presenting problem, compared to 68% who accurately diagnosed depression. There were no significant differences in diagnostic accuracy across professional disciplines. However, psychiatrists had significantly greater knowledge of symptoms compared with the other professional groups, and, along with psychologists, endorsed the greatest level of confidence in diagnosing and managing BED.

Supina et al. [55] undertook a cross-sectional survey of 278 physicians in the US, including psychiatrists (23%), family practitioners (27%), internists (31%), and OB-GYN (19%). Participants completed an online survey in which they were first presented with case vignettes depicting DSM-5 BED, followed by an assessment of diagnostic, screening, and referral practices. After reviewing the BED vignette, 92% of physicians reported they would be “very likely, likely, or somewhat likely” to assess for an ED. Of these participants, 74% (*N* = 206) then correctly identified BED as the accurate ED diagnosis. A majority reported relying upon clinical history (97.1%) to establish a diagnosis of BED, whereas less than one-third would use the full DSM-5 criteria, and only 13.6% would use a screening instrument. Approximately one-third did not recognize BED as a discreet ED diagnosis. Frequency of binge eating (84%) and degree of impairment (75.2%) were cited as BED characteristics used to determine clinical severity. Of the 172 physicians that had reported ever treating BED, 59% reported they would “always” or “frequently” refer to a dietician or recommend exercise (56.4%). A little less than half would recommend cognitive-behavioral therapy, and approximately one-third would recommend pharmacological treatment, behavioral weight control, weight watchers (or similar), or interpersonal therapy. Approximately 22% would recommend guided self-help, and 10% would recommend pure self-help. 

Lastly, a study by Cain et al. [52] investigated practitioners’ knowledge and attitudes toward BED among “frontline” healthcare professionals in Australia. This included general practitioners (48.5%), clinical dieticians (21.7%), medical students (9.7%), and others (20.1%; nurses, psychologists, psychiatrists, and registrars). Participants (*N* = 175) were randomly assigned to either a BED or obesity-only condition (BMI = 35.0 in both vignettes) and then completed items related to diagnostic knowledge, physical complications, and treatment alternatives. Over 80% correctly identified BED as the presenting problem and 77.1% correctly recognized obesity-only. However, only a quarter of participants (26.6%) in the BED condition also correctly recognized the comorbid presence of obesity. Further, participants in the BED condition had less knowledge of potential physical complications than those in the obesity-only condition, despite the similar BMI. Approximately half of the participants in the BED condition recognized all four provided diagnostic criteria for BED (recurrent episodes of binge eating, a sense of lack of control over eating, feeling disgusted with oneself, depressed or guilty afterwards, recurrent episodes 1x a week for 3 months). Eighty-seven percent endorsed CBT as a treatment option for BED, followed by selective serotonin reuptake inhibitors SSRIs (38%), interpersonal therapy (30%), behavioral weight loss (29%), and psychodynamic therapy (27.8%).

## 5. Discussion

The present review investigated the emerging research on lay and healthcare professionals’ knowledge and attitudes toward BED. Until recently, research has focused primarily on anorexia nervosa or bulimia nervosa [31,32,33,66,67,68,69,70], yet the inclusion of BED as a formal psychiatric diagnosis as an eating disorder in the DSM-5 [1] renders an examination of this issue timely. 

### 5.1. Summary of Community Studies

A total of 13 studies were reviewed, including nine community studies and four studies of various healthcare professionals. A lack of self-discipline or self-control was perceived to contribute more to the development of BED than illnesses such as depression or Type 1 diabetes [57,60]. Moreover, strength in the belief that a “lack of self-discipline” was causal was associated with more stigmatizing attitudes [58]. Similar to other EDs, individuals with BED encounter negative societal stereotypes reflective of perceived blameworthiness. Yet, the extent to which perceived blameworthiness facing BED exceeds that for AN or BN is less clear. In the study by Ebneter & Latner (2013) [57], the BED target was blamed more than AN or BN, whereas another study found similar levels of blameworthiness across the EDs [63]. More consistently, individuals with BED were perceived as less impaired, easier-to-treat, and “more able to pull themselves together” than the other EDs [57,61,63]. Notably, several studies found that male respondents held more stigmatizing attitudes than female respondents [56,61,64].

Two community studies demonstrated a surprisingly low level of public awareness that BED was a discreet eating disorder [59,60]. An earlier Australian population study found that the public perceived binge eating as a problem of depression or low self-esteem, rather than an eating disorder per se [59], and none of the high school students in an Irish study accurately recognized BED [60]. More recently, a higher proportion of adolescents in Australia correctly recognized BED than BN, yet this percentage was only one-third for boys and 43% for girls [64]. While it is encouraging that half of the adolescents in that study agreed that BED was serious and required professional treatment, this proportion was lower compared to BN [64]. Another study found that high school students ascribed fewer positive personality traits to BED than to major depression or AN [60]. Poor awareness and negative attitudes toward BED by younger persons is particularly worrisome, as this cohort is at-risk for the development of EDs, which tend to track over time, and attitudes held by their peers are considered pivotal for social support or stigmatization [60]. 

Collectively, this body of literature has several methodological shortcomings which limit the conclusions which can be drawn for BED. Nearly all of the case vignettes for BED depicted a young and female character. Further, despite not specifying racial or ethnic background, a non-Hispanic white ethnicity might have been inferred from the names of the fictional characters (e.g., Ashley, Emily, Jenny). Future studies of vignette-based methodology should incorporate more balanced representation of BED vignettes, which would also enable analysis of attitudes toward and recognition of BED in males and racial/ethnic minority groups. This is a particularly salient methodological issue because the gender ratio of BED is estimated at 6:4 for females to males, and BED is as common among ethnic minority and racial groups as among non-Hispanic whites [1].

It remains unclear the extent to which the presence of BED might differentiate, or add incrementally to, stigmatizing attitudes encountered by individuals with obesity. Although few studies specified exact BMIs in the case descriptions, all of the BED vignettes were described as “overweight” or as having difficulties with weight management. People with obesity face pervasive societal exposure to weight discrimination [71], as well as self-directed, or internalized, weight stigma [72]. Clinically, weight-related stigma has deleterious effects on health and well-being, as well as the exacerbation or perpetuation of binge eating or other forms of maladaptive eating [29,50,73]. Of the few studies directly comparing obese vignettes with or without binge eating, one study found that the obese vignette with binge eating was rated as less attractive, having a worse prognosis, and more blameworthy [56]. Yet another study found no differences in the level of blameworthiness between obese individuals or without BED [63], and one study found the opposite effect [57]. In one study, societal discrimination toward individuals with comorbid obesity and BED was widely anticipated; however, the vast majority (84%) in a community study believed that discrimination would be weight-related rather than eating-related [62]. As such, existing studies fail to discern clearly between sources of stigma. Future studies are suggested to also include normal-weight presentations of BED to allow for a more nuanced investigation of this issue. 

### 5.2. Summary of Healthcare Professional Studies

Literature on healthcare professional knowledge and attitudes toward BED remains very limited. The relatively few studies suggest encouraging trends toward improving recognition and diagnostic accuracy, yet there remains a need for increased awareness of BED-associated comorbidities and knowledge of full diagnostic criteria among healthcare practitioners. The 11th Revision of the International Classification of Diseases (ICD-11) is due by 2018 and is expected to follow the DSM in the recognition of BED [74], which should boost recognition worldwide. A total of four studies were reviewed from US, Ireland, and Australia [52,53,54,55]. Collectively, these studies diverged in findings, with two studies reporting rather low rates of assessment (i.e., 40% of physicians had never assessed for BED) [53] and diagnostic accuracy (i.e., 19% correctly diagnosed BED) [54] and two studies offered encouraging findings, with the vast majority of professionals correctly identifying BED [52,55]. Divergent findings are likely attributable to methodological issues, such as the very low response rate of 9% in the Irish study [54], and the fact that the study by Crow et al. utilized an unspecified method to estimate binge eating. The study by Crow et al., however, may provide an approximate baseline of screening and referral practices prior to the inclusion of BED in the DSM-5, as the study was conducted in 2004 [53].

In terms of referral and treatment practices, the Australian study found that 87% of professionals recommended CBT as a treatment [52] compared to slightly less than half of physicians in the US study [55]. Additionally, 59% of US physicians reported they would “always” or “frequently” refer to a dietician or recommend exercise (56%) [55], whereas approximately one-third of professionals recommended behavioral weight loss, interpersonal therapy, SSRIs [52], or pharmacotherapy [55]. Of note, only one-quarter of healthcare professionals accurately recognized the presence of obesity when co-occurring with BED [52], despite being shown a vignette with a BMI of 35. Moreover, participants demonstrated less knowledge of physical complications when BED accompanied obesity. For instance, nearly 100% of participants recognized high blood pressure in the obesity-only condition, but this rate dropped to 60% when BED was present, despite the similar BMIs. 

As many patients with BED may be motivated to seek treatment due to physical complications [75], there remains a need for increased awareness of BED-associated comorbidities among healthcare practitioners [25]. Future investigations should continue to address the effects of a complex comorbid presentation of BED and obesity on screening, assessment, referral, and treatment practices. It is also important to evaluate the influence of knowledge and attitudes toward BED on treatment decisions, akin to prior research with AN [76], and to investigate clinical behavior beyond fictional case vignettes. Low response rates and representativeness are methodological issues which should be addressed going forward, and a broader cross-section of professionals who are likely to encounter BED should be assessed, including teachers and school counselors.

## 6. Conclusions

This review summarized the emerging literature on lay and healthcare professionals’ knowledge and attitudes toward BED to inform future mental health awareness campaigns, stigma reduction efforts, clinical practice, and further research. Several main findings can be drawn from the emerging literature. First, despite being recognized as a distinct psychiatric illness in the DSM-5 [1], there remains a low level of public recognition and awareness of BED. Second, although BED is associated with significant role impairment, as well as medical and psychiatric comorbidity, BED is perceived in the community as less impairing and less severe than other EDs. Third, individuals with BED are subject to societal perceptions of blameworthiness and perceived controllability of their illness. Moreover, poor willpower or lack of self-discipline is perceived to contribute more to the development of BED than other illnesses. Fourth, males appear to hold more stigmatizing attitudes toward BED than females. Lastly, although the level of knowledge and recognition of BED by healthcare professionals appears to be increasing, in particular by psychiatrists, very little remains known regarding actual screening, assessment, referral, and treatment practices. 

Future research should include greater diversity in vignettes to adequately reflect the gender, ethnic distribution, and racial distribution of BED. Additionally, increased specificity is warranted to differentiate or detect incremental effects of stigma that are likely associated with overweight/obesity versus BED specifically. This is an important issue, given the enormity of the weight of stigma and discrimination in society, coupled with the distinct and deleterious effects of weight stigma on eating behavior [29,50,73]. Moving forward, stigma reduction interventions for BED would benefit from educational elements garnered from research on stigma and obesity, as weight bias is a concept that is important for well-being across eating and weight-related issues [77,78,79,80]. To date, public awareness campaigns and stigma reduction interventions have focused primarily upon AN, or EDs generally [27], and tailored efforts for BED are sorely needed. 

## Figures and Tables

**Table 1:** Study characteristics of investigations of community attitudes and knowledge of binge eating disorder (BED).

Study	Country	*N*	Sample	Method	Case Description	Main Findings
[59]	Australia	1031	Community	Case vignette	32-year old female with binge eating behavior and obesity	Only 11.7% viewed an eating problem as “main” px Two-thirds felt either depression or low self-esteem was the “main” px 4.6% felt CBT would be most helpful treatment
[56]	USA	376	University students	Case vignette	Female with obesity (each with 3 causal scenarios: biological, psychological, or ambiguous): With binge eating Without binge eating	Vignette with obesity + BE rated more negatively, having a worse prognosis, less attractive, more blameworthy Participants desired greater social distance from vignette with obesity + BE Male respondents held more stigmatizing attitudes against the vignette with obesity + BE
[58]	USA	447	University students	Case vignette	19-year old female with: AN BN BED (“overweight”) Obesity	Stigma was associated with strength of belief in “just world” and causal attribution to “lack of self-discipline” Participants who knew someone with BED did not differ in level of stigma compared to those who did not
[57]	USA	447	University students	Case vignette	19-year old female with: AN BN BED (“overweight”) Obesity Depression	Vignette with BED was blamed more than other EDs or depression Vignette with BED was considered less impaired than other Eds or depression Lack of self-discipline was perceived to contribute more to obesity and BED Target with comorbid obesity-BED was held less personally responsible than target wtih obesity only
[62]	Australia	3047	General population	Case vignette	AN, female, underweight Atypical ED, male, normal wt BED, female, obesity	66% believed the BED/obesity target would be discriminated against Of these, 84% believed discrimination would be weight-based, not due to ED
[64]	Australia	1670	Adolescents	Case vignette	15-year old female with BN BED (“overweight”)	About 30% of boys and 42% of girls recognized BED as the main problem One-third of boys and 20% of girls felt BED was a problem of “lack of will-power/self-control” Fewer agreed BED was serious and required professional help vs. BN Lower perceptions of severity found among boys than girls
[63]	USA	318	University students	Case vignette	Male and female versions: AN BN BED (unknown BMI) Obesity	Targets with BED were found similarly blameworthy as AN, BN and obesity BED was rated as less impaired than AN and BN but more impaired than obesity-only BED was more positively judged than BN, and elicited less discomfort
[60]	Ireland	290	High school students	Case vignette	Gender-neutral AN BN BED (“overweight”) Depression Type 1 diabetes	None of the participants correctly categorized BED BED was causally attributed to “self-control problems” by 41% of participants (vs. 1.9% for BN and 1.4% for MDD) BED was ascribed less positive personality traits than MDD or AN More positive reactions anticipated to interacting with BED than MDD
[61]	USA	505	University students	Case vignette	19-year old female with: AN BN BED (“overweight”) Orthorexia	BED viewed as less “dangerous” and “easier to talk to” than AN or orthorexia BED viewed as more able to “pull themselves together if they wanted to” than orthorexia BED would be easier to treat than AN or BN BED less influenced by biological factors than AN or orthorexia Male respondents perceived all EDs to be less severe, acting out of need for attention, and more at fault for their condition than females

AN = anorexia nervosa; BN = bulimia nervosa; BED = binge eating disorder; BE = binge eating; CBT = cognitive-behavioral therapy; MDD = major depressive disorder; px = problem.

**Table 2:** Study characteristics of investigations of healthcare professionals’ attitudes and knowledge of BED.

Study	Country	*N*	Sample	Method	Case Description	Main Findings
[53]	USA	272	Healthcare professionals	Mail-out survey	N/A	BED never assessed by 40% of physicians An additional 42.8% estimated that 0 to 20% of their patients with obesity engaged in binge eating
[54]	Ireland	171	Healthcare professionals	Case vignette	15-year old gender-neutral: AN BN BED (“overweight”) Depression Type 1 diabetes	BED correctly diagnosed by 19% of healthcare professionals Psychiatrists had higher mean levels of symptom knowledge than other medical disciplines
[55]	USA	278	Healthcare professionals	Case vignette	Case #1 a. 32-year old female with BED and overweight If participants responded “don’t know” or “not likely/would not assess, they were shown: Case #2 b. 46-year old male with BED and obesity	92% were “very likely, likely, or somewhat likely” to assess for an ED Of these, 74% correctly identified BED Majority relied upon clinical history (97%), fewer would use the full DSM-5 criteria (29.6%) or an instrument (13.6%) to establish dx 46% would recommend CBT
[52]	Australia	175	Healthcare professionals	Case vignette	19-year old female: BED-obesity Obesity	82.3% correctly identified BED Only 26.6% in BED condition recognized comorbid obesity Less knowledge of physical complications in BED 87% endorsed CBT as treatment option for BED

AN = anorexia nervosa; BN = bulimia nervosa; BED = binge eating disorder; BE = binge eating; CBT = cognitive-behavioral therapy; DSM-5= diagnostic and statistical manual-5th edition; dx = diagnosis; N/A = not applicable.
